# Stable transmission of an unbalanced chromosome 21 derived from chromoanasynthesis in a patient with a *SYNGAP1* likely pathogenic variant

**DOI:** 10.1186/s13039-018-0394-0

**Published:** 2018-08-28

**Authors:** Peter J. B. Sabatini, Resham Ejaz, Dimitri J. Stavropoulos, Roberto Mendoza-Londono, Ann M. Joseph-George

**Affiliations:** 1 Laboratory Medicine Program, Department of Pathology, University Health Network, 200 Elizabeth St, Toronto, ON M5G 2C4 Canada; 2 Department of Laboratory Medicine and Pathobiology, University of Toronto, Toronto, ON Canada; 3 Division of Clinical and Metabolic Genetics, Department of Paediatrics, The Hospital for Sick Children, University of Toronto, Toronto, ON Canada; 4 Genome Diagnostics, Department of Paediatric Laboratory Medicine, The Hospital for Sick Children, University of Toronto, Toronto, ON Canada

**Keywords:** Chromoanasynthesis, *SYNGAP1*, Familial transmission, Chromoanagenesis

## Abstract

**Background:**

Complex genomic structural variations, involving chromoanagenesis, have been implicated in multiple congenital anomalies and abnormal neurodevelopment. Familial inheritance of complex chromosomal structural alteration resulting from germline chromoanagenesis-type mechanisms are limited.

**Case presentation:**

We report a two-year eleven-month old male presenting with epilepsy, ataxia and dysmorphic features of unknown etiology. Chromosomal microarray identified a complex unbalanced rearrangement involving chromosome 21. G-banding and FISH for targeted regions of chromosome 21 revealed that the copy number imbalances were limited to gains dispersed throughout the long arm of chromosome 21, characteristic of a chromosome derived from chromoanagenesis. Family studies showed that the unbalanced chromosome had been stably inherited, as it was present in both his healthy mother and maternal grandfather. Further molecular testing for non-syndromic intellectual disability genes found a likely pathogenic mutation in *SYNGAP1* (NM_006772.2:c.3722_3723del).

**Conclusions:**

This study indicates that complex rearrangements involving an unbalanced chromosome derived from chromoanasynthesis can be familial and should be not be presumed pathogenic.

## Background

Chromoanagenesis is a class of genomic structural variation that is identified by alternating copy number states across a single chromosome or confined to specific loci of one or more chromosomes [1]. Based on the proposed repair mechanisms by which they are generated, two main sub-categories of chromoanagenesis are recognized. Chromothripsis refers to a shattering event that can involve multiple chromosomes, massive DNA losses and over 10 breakpoints. In contrast, chromoanasynthesis is usually confined to a single chromosome and typically restricted to specific loci containing genomic gains [2].

Healthy carriers of balanced rearrangements arising from chromoanagenesis can transmit unbalanced derivatives to offspring [3, 4]. These families were identified cytogenetically as having either classic reciprocal translocations or more complex rearrangements; however, sequencing of the breakpoints show pieces of alternating chromosomes inserted into the breakpoints. Other case reports show that balanced carriers of these structural variations can also be affected when the breakpoints disrupt known disease-causing genes [5, 6].

Given the severity of the imbalances and multiple breakpoints resulting from chromoanagenesis, the discovery of such findings in a diagnostic setting could support pathogenicity. Here we report a derivative chromosome 21 that displays the classic features of chromoanagenesis with numerous gains across the entire long arm of chromosome 21 in a child with global developmental delay (GDD), epilepsy and ataxia. The derivative chromosome was stably inherited from the child’s healthy mother and maternal grandfather. Upon further genetic testing, the child was shown to have a likely pathogenic variant in *SYNGAP1* consistent with his clinical features. This is the first report to describe a stably inherited unbalanced derivative chromosome 21 resulting from chromoanasynthesis. The diagnostic challenge of over-interpreting such findings show the importance of family studies and assessing clinical context.

## Case presentation

The patient was a two-year eleven-month old boy born to non-consanguineous 21-year-old primigravida mother of Ashkenazi Jewish descent and 25-year-old father of Ashkenazi Jewish and Irish descent. Pregnancy was uncomplicated and he was born at 41 + 4 weeks gestation via spontaneous vaginal delivery. Apgar scores were 9 at 1 and 5 min. Birth weight was 3.834 kg (50-75th centile). Neonatal course was unremarkable.

Developmental concerns arose in early infancy. He sat unsupported after twelve months of age and walked independently at twenty-two months of age. At 2 years, 3 months of age, he could go upstairs with two-hand support and climb furniture. Gait was ataxic. He had a palmer grasp, could hold objects at the midline for thirty seconds, and could not transfer objects. He did not have any words but had recently started using gestures. Reception was limited to one step commands. He was easily excitable but demonstrated good socialization attempts with other children.

Medical history was significant for myoclonic seizures starting between 2 to 3 years of age, requiring anti-epileptic medications. He preferred pureed foods, with occasional choking episodes. He also displayed preference for specific textures, and a fascination for water. Sleep was disrupted with frequent awakenings, thought to be behavioural. Echocardiogram, abdominal ultrasonography, brain magnetic resonance imaging (MRI), and genetic testing for Angelman syndrome were normal.

**Fig. 1:**
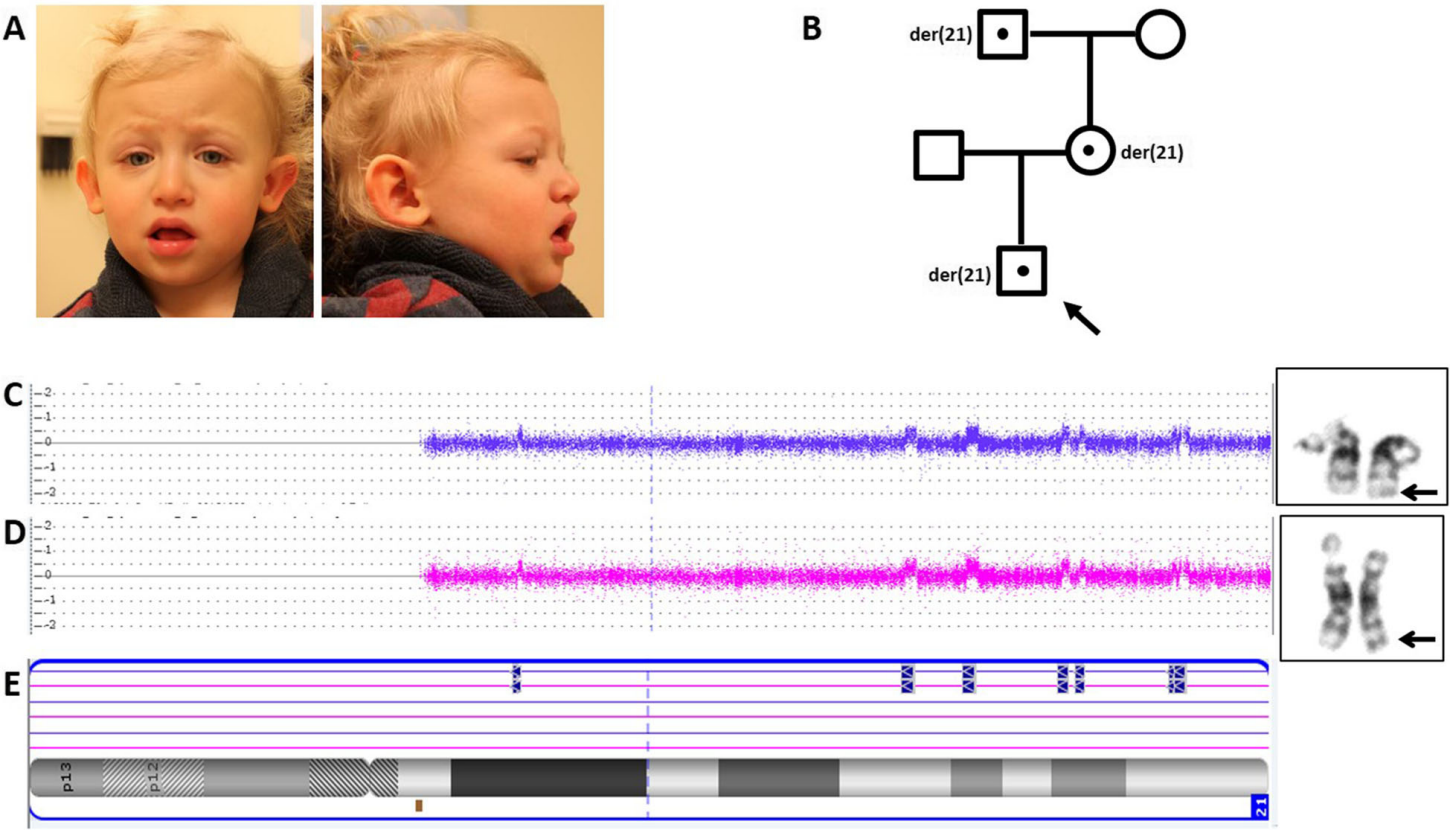
**a** Images of proband, **b** Pedigree displaying family members who carry the unbalanced der(21). Results of the mother (**c**) and proband (**d**) showing genomic microarray log_2_ ratio plots and partial karyotypes of chromosome 21 with the derivative (arrow), **e** Ideogram of chromosome 21 showing genomic regions of copy number gains in both individuals

At 2 years, 3 months of age, weight was 12.1 kg (15-50th centile), height was 87.5 cm (15-50th centile) and head circumference was 48 cm (− 1 SD). He had deep-set eyes, down slanting palpebral fissures, prominent nasal root and tip, prominent ears, with a myopathic expression (Fig. 1a). An intention tremor and ataxic gait were noted.

Family history was notable for maternal anxiety, and attention deficit disorder in two maternal cousins. His mother and maternal grandfather had completed college and post-graduate professional education respectively. There was no family history of GDD, ataxia, other neurological disorders, infertility or multiple miscarriages. As the father was no longer involved in the child’s care, further detailed paternal history was unavailable.

## Methods

### Microarray

Microarray analysis was performed using DNA extracted from peripheral blood using the Affymetrix CytoScan HD Array. Copy number changes were analyzed using the Chromosome Analysis Suite software (Affymetrix).

### G-band and FISH analyses

Peripheral blood specimens for chromosome analysis were cultured, harvested and prepared for G-band and Fluorescence in situ hybridization (FISH) using standard protocols. FISH analyses was carried out using whole chromosome paint 21 (ID Labs), subtelomeric 21qTel (Cytocell) and labelled BAC clones for chromosome 21: RP11-1150I14, RP11-70F1, RP11-483 M4, RP11-360 N24, RP11-137 J13, RP11-146O16, RP11-268D8 (The Centre for Applied Genomics, Toronto, Canada) with Metasystems software.

Microarray, FISH and G-banding studies were performed on the proband and mother. Further family studies were done by FISH and G-banding on the maternal grandfather. Informed consent was obtained from all parties prior to clinical testing.

## Results

### Chromosome microarray

Genomic microarray analysis detected seven copy number gains confined to chromosome 21 ranging from 147 to 461 Kb in size. These encompassed 36 RefSeq genes and 5 OMIM morbid genes, *CRYAA*, *SIK1*, *C21orf59*, *SYNJ1,* with one gain partially overlapping the *RUNX1* locus. All gains were classified uncertain clinical significance. The genomic coordinates of these regions are: arr[hg19]21q21.1(18808979_18965672)× 3,21q22.11(33881877_34331853)× 3,21q22.12(36267228_36727932)× 3,21q22.2(39968481_40301704)× 3,21q22.2(40648264_40885013)× 3,21q22.3(44288908_44436177)× 3,21q22.3(44539705_44928148)× 3. Family studies using FISH revealed consistent gains in both the patient’s mother (Fig. 1b) and maternal grandfather.

**Fig. 2:**
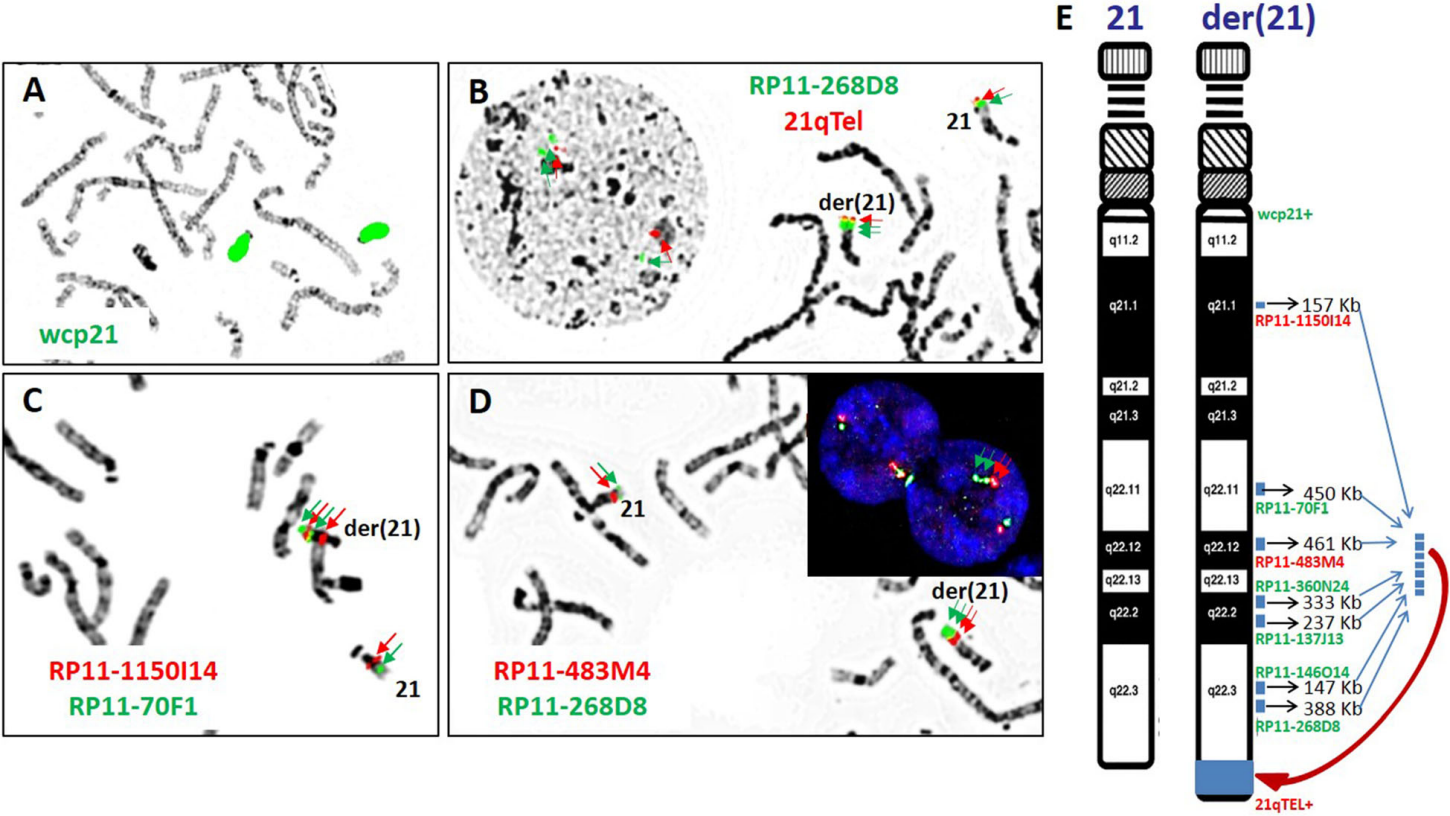
FISH results of the proband using BAC probes shown in ideogram: (**a**) whole chromosome paint 21, **b** RP11-268D8 and 21qTel, **c** RP11-1150I14 and RP11-70F1 and (**d**) RP11-483 M4 and RP11-268D8. **e** Schematic illustration of rearrangement observed in derivative chromosome 21. The FISH results suggest the copy number gains aggregate at the distal long arm of the derivative chromosome 21

G-banding and FISH follow-up were completed to characterize the rearrangement. A derivative chromosome 21 with additional chromosomal content at the terminal region of its long arm was detected by G-banding in both the patient and his mother (Fig. 1c and d). FISH studies on the proband using BAC probes targeted to all regions of gain (RP11-1150I14, RP11-70F1, RP11-483 M4, RP11-360 N24, RP11-137 J13, RP11-146O16, RP11-268D8) and subtelomeric 21qTel probe showed a discrete signal for probes at their expected locations, on both the derivative and normal chromosomes 21. Additional FISH signals for the gained segments appeared translocated and clustered at the distal region of the long arm of the der(21), inserted just proximal to the 21q subtelomeric probe. Although the gained material appeared to be reconstituted together (Fig. 2e), neither the orientation nor relative organization of the segments relative to each other were evident.

### Molecular testing

Given the generational inheritance of the derivative chromosome 21 including unaffected individuals, another genetic etiology was suspected. A non-syndromic intellectual disability panel at a Clinical Laboratory Improvement Amendments (CLIA)-certified laboratory revealed a likely pathogenic 2 base pair deletion in exon 17 of *SYNGAP1* (NM_006772.2:c.3722_3723del; p.Leu1241Argfs*4), predicted to result in a premature stop codon 4 positions downstream. The variant occurs in the SYNGAP1 coiled-coils (CC) domain [7]. This deletion had not been previously described in normal population databases. Other frameshift truncating pathogenic variants in *SYNGAP1* have been described in individuals with moderate to severe intellectual deficiency (ID), ataxia, and epilepsy [8]. The variant was not present in the mother through targeted testing and paternal sample was not available for testing.

## Discussion

Here we describe a case of a stably transmitted derivative chromosome 21 resulting from a complex intra-chromosomal rearrangement with features characteristic of chromoanasynthesis. The proband also harboured a likely pathogenic variant in *SYNGAP1* that fits his phenotype. These results show that comprehensive ascertainment of differential diagnoses should be followed-up even in the context of rare unbalanced microarray findings. The study also confirms that healthy individuals can be carriers of unbalanced, likely benign, complex structural changes resulting from chromoanasynthesis-type of rearrangements.

Highly complex genomic rearrangements known collectively as chromoanagenesis do not arise from sequential accumulation of independent rearrangements, rather they are thought to occur spontaneously as a single event [1, 9]. Two categories of constitutional chromoanagenesis have been observed. Chromoanasynthesis describes a complex structural rearrangement involving a single chromosome and usually restricted to predominantly copy number gains. In contrast, chromothripsis includes losses, gains and retention of copy neutral states that tend to localize to confined genomic regions. Our family displays features of chromoanasynthesis based on the accumulation of seven copy numbers gains confined to chromosome 21. One study describes a similar distinct class of chromoanagenesis characterized by focal copy number gains containing regions of microhomology, double stranded blunt ends and non-templated inserted sequences at the breakpoints [10].

Most congenital complex chromosomal structural variations occur de novo. The origins of chromoanagenesis are still unknown; given its occurrence in both congenital disorders and cancer, the initiating event can occur as meiotic error during gametogenesis or mitotic instability [2, 11–13]. A primary event for our patient’s familial rearrangement could not be traced. Previous studies have described unbalanced rearrangements arising from chromosome shattering and repair in affected patients with variable phenotypes. Complex structural variations, including chromoanagenesis, have been seen in individuals with autism spectrum disorder (ASD), GDD, and dysmorphic facial features [6]. They have also rarely been seen in families with phenotypically unaffected individuals, with an increased risk of having children with congenital anomalies or recurrent miscarriages due to unbalanced rearrangements [3, 5]. Interestingly, our family did not have any history of recurrent miscarriages or congenital anomalies with eight children fathered by the patient’s grandfather. Chromoanasynthesis events specifically confined to copy number gains in chromosome 21 have not been reported. In addition, reports of reconstitution of all the gains together and their intrachromosomal insertional translocation as a cluster within the derivative chromosome appear to be extremely rare (patient BAB3105 in [12]).

Heterozygous pathogenic variants in *SYNGAP1* were first described in three of ninety-four individuals with non-syndromic intellectual disability (ID), predicted to be related to the gene’s role in synaptic plasticity [7]. Since then, several reports have expanded the gene-related phenotype to include ASD, epilepsy, frequently characterized by myoclonic or atonic seizures, and ataxia [14, 15]. It is estimated that pathogenic variants in the gene may account for up to 1% of epileptic encephalopathies and ID each [8, 15]. Descriptions of shared facial and clinical features between affected individuals have also suggested a recognizable pattern for a *SYNGAP1*-associated syndrome [16]. Our patient’s presentation, including his myopathic facial features, ataxia, global developmental delay, epilepsy, and sleep, is consistent with the previously reported *SYNGAP1* phenotype.

## Conclusions

This report describes the presence of a stably inherited derivative chromosome 21 displaying features of chromoanasynthesis over at least 3 generations. The derivative chromosome 21 was found in an individual with global developmental delay, epilepsy and ataxia; however, it was also found in two unaffected family members. The proband was subsequently found to have a *SYNGAP1* likely pathogenic variant as the underlying etiology for his phenotype. By not pursuing family studies and investigating alternative genetic mutations related to the phenotype, the child’s unbalanced rearrangement could have been erroneously correlated to his presentation. Our report adds to the growing literature of stable inheritance of complex chromosomal rearrangements, emphasizing that rarity does not necessarily imply causality.
